# Prolonged Sitting Time: Barriers, Facilitators and Views on Change among Primary Healthcare Patients Who Are Overweight or Moderately Obese

**DOI:** 10.1371/journal.pone.0125739

**Published:** 2015-06-09

**Authors:** Elena Martínez-Ramos, Carme Martín-Borràs, José-Manuel Trujillo, Maria Giné-Garriga, Carlos Martín-Cantera, Mercè Solà-Gonfaus, Eva Castillo-Ramos, Enriqueta Pujol-Ribera, Dolors Rodríguez, Elisa Puigdomenech, Angela-Maria Beltran, Noemi Serra-Paya, Ana Gascón-Catalán, Anna Puig-Ribera

**Affiliations:** 1 Primary Healthcare Centre, Vilanova 1, Institut Català de la Salut (ICS), Barcelona, Spain; 2 Lifestyles Study Group, RedIAPP, Institut Universitari d'Investigació en Atenció Primària Jordi Gol (IDIAP Jordi Gol), Barcelona, Spain; 3 Department of Physical Activity and Sport Sciences, FPCEE Blanquerna, Universitat Ramon Llull, Barcelona, Spain; 4 Primary Healthcare Centre, Cuevas del Almanzora, North Almeria Region, Spain; 5 Department of Physical Therapy, FCS Blanquerna, Universitat Ramon Llull, Barcelona, Spain; 6 Department of Medicine, Universitat Autònoma de Barcelona, Bellaterra (Cerdanyola del Vallès), Barcelona, Spain; 7 Primary Healthcare Centre, Les Planes, ICS, Barcelona, Spain; 8 Primary Healthcare Centre, Molí Nou, ICS, Barcelona, Spain; 9 Institut Català de la Salut, Generalitat de Catalunya, Barcelona, Spain; 10 Department of Nursing, Universitat de Lleida, Lleida, Spain; 11 Department of Health Sciences, University of Zaragoza, Zaragoza, Spain; 12 Grup de Recerca en Esport i Activitat Física. Universitat de Vic. Universitat Central de Catalunya (UVic-UCC), Vic, Spain; University of Alabama at Birmingham, UNITED STATES

## Abstract

**Background and Objectives:**

Prolonged sitting time has negative consequences on health, although the population is not well aware of these harmful effects. We explored opinions expressed by primary care patients diagnosed as overweight or moderately obese concerning their time spent sitting, willingness to change, and barriers, facilitators, goals and expectations related to limiting this behaviour.

**Methods:**

A descriptive-interpretive qualitative study was carried out at three healthcare centres in Barcelona, Spain, and included 23 patients with overweight or moderate obesity, aged 25 to 65 years, who reported sitting for at least 6 hours a day. Exclusion criteria were inability to sit down or stand up from a chair without help and language barriers that precluded interview participation. Ten in-depth, semi-structured interviews (5 group, 5 individual) were audio recorded from January to July 2012 and transcribed. The interview script included questions about time spent sitting, willingness to change, barriers and facilitators, and the prospect of assistance from primary healthcare professionals. An analysis of thematic content was made using ATLAS.Ti and triangulation of analysts.

**Results:**

The most frequent sedentary activities were computer use, watching television, and motorized journeys. There was a lack of awareness of the amount of time spent sitting and its negative consequences on health. Barriers to reducing sedentary time included work and family routines, lack of time and willpower, age and sociocultural limitations. Facilitators identified were sociocultural change, free time and active work, and family surroundings. Participants recognized the abilities of health professionals to provide help and advice, and reported a preference for patient-centred or group interventions.

**Conclusions:**

Findings from this study have implications for reducing sedentary behaviour. Patient insights were used to design an intervention to reduce sitting time within the frame of the SEDESTACTIV clinical trial.

## Introduction

“Sedentary behaviour” encompasses all those activities carried out while sitting (reading, sewing, watching television, and other forms of on-screen entertainment) and that involve a very low energy use (1–1.5 metabolic equivalent of tasks, METS) [1]. In today’s society, sedentary activities have replaced a large portion of the time that used to be dedicated to light physical activity, such as standing or walking [2], and adults spend 51% to 68% of their waking hours sitting [3–5].

Sedentary behaviour has negative health consequences [6–9], being associated with chronic illnesses such as obesity, alterations in glucose metabolism and diabetes mellitus type II, metabolic syndrome, osteoporosis, and some cancers [3, 10]. Prolonged sitting time is also associated with increased mortality, especially due to cardiovascular disease, and this association is independent of the level of physical activity [11–13].

There is controversy about the number of sedentary hours per day that are prejudicial to health. Some studies have found a higher mortality rate among individuals who are seated for 6 or more hours a day, compared to those who spend fewer than 3 hours a day sitting [11]; others have reported a greater mortality risk in those seated for more than 4 hours [3]. A recent study has shown that reducing the time spent sitting by at least 3 hours a day can increase life expectancy by 2 years [10].

Sedentary behaviour can coexist with different patterns of physical activity [14]. On the same day, it is possible to sit for a prolonged time and also participate in the amount of physical activity recommended for health, or do very little physical activity but not spend much time sitting. Evidence shows that these two behaviours are independent, with different health consequences [2, 11–13, 15, 16]. In addition, eating behaviours are common during many sedentary activities (watching television, going to the cinema, reading), which increases the probability of weight gain [17].

Obesity is considered the “epidemic” of the twenty-first century. People who are overweight or obese do less physical exercise and spend more time each day sitting [16, 18]. Current intervention for obesity and overweight is based on diet, physical exercise and psychological support [19]. However, this is a complex phenomenon, and interventions have limited long-term efficacy because of low adherence over time [20]. A recent study by Healy et al. observed that a reduction in sitting time can improve the metabolic consequences of obesity, regardless of the level of activity [4].

Research on the adverse health effects of remaining seated for prolonged periods of time is rather new. Therefore, it is probable that in many cases neither patients nor their primary healthcare professionals are conscious of the problem and its consequences. In addition, only a few randomized, controlled clinical trials have evaluated the impact of interventions to reduce daily sitting time [21–26]. For these reasons, it is necessary to design primary healthcare interventions with this aim that are feasible, practical, acceptable, and effective, directed especially toward individuals who are overweight or obese. To implement programs based on these multi-component interventions and improve patient adherence, it is important to understand what sedentary behaviour means for the target population.

The aim of this study was to look in depth at the opinions of overweight or moderately obese patients who sit for prolonged periods of time each day concerning ways to reduce or limit this behaviour, considering their willingness to change, the barriers and facilitators, and the prospects of receiving help from primary healthcare professionals.

Specifically, the study analysed participants’ opinions and beliefs regarding the time that is spent sitting (at work and during free time), their willingness (based on importance, motivation and confidence) to make changes and suggestions on how to reduce this behaviour.

## Methods

### Study design

This qualitative descriptive-interpretive study was framed within a larger project, entitled “Effectiveness of a primary care-based intervention to reduce sitting time in overweight and obese patients (SEDESTACTIV): a randomized controlled trial” and financed by the Spanish government’s *Fondo de Investigación Sanitaria* (PI11/01082) [27]. The results of this qualitative study were essential in defining the rationale and study design for the SEDESTACTIV clinical trial intervention.

Consolidated criteria for reporting qualitative studies (COREQ) guidelines were used to design and conduct the study [28]. Qualitative methodology was chosen because it was considered to be the most appropriate to achieve a deeper understanding of subjective and complex phenomena such as the factors that explain and interact in sedentary behaviours.

### Ethics statement

The study protocol was approved by the Clinical Research Ethics Committee of the Jordi Gol Research Institute for Primary Care. Written informed consent was obtained from all patients prior to participation. The study was performed in accordance with the declaration of Helsinki II [29].

### Participants

Study participants were recruited from three primary healthcare centres (PHC) in the Barcelona area and surroundings. Inclusion criteria were the following: (a) aged 25–65 years, (b) diagnosed as being overweight or moderately obese (body mass index, BMI ≥ 25–34.9 kg/m^2^) and (c) daily spend 6 or more hours sitting, as reported on the Marshall questionnaire [30]. Individuals were ineligible for the study if they were not independent in sitting down or standing up from a chair, had undergone obesity surgery, or did not understand Spanish or Catalan sufficiently to participate in the interview.

A convenience sample was selected from patients who visited the healthcare centre, fulfilled the inclusion criteria and agreed to participate. To ensure a wide variety of discourses on sedentary behaviour, selected participants were of both sexes, of different ages and occupations, and had different levels of overweight and moderate obesity. The number of participants (*n* = 23) and number of interviews (*n* = 5 group and 5 individual interviews) were determined by information saturation. Before beginning the individual and group interviews, the objectives of the study were explained, along with the length of the interviews, expected uses of the information obtained, need to audio record the interviews, and assurances of confidentiality. Informed consent was obtained from all participants.

Participant characteristics and the number of participants for each interview technique are presented in Table 1.

**Table 1:** Summary of participant characteristics.

Interview type	Sex	Age (years)	Time (hours)	BMI (kg/m^2^)	Occupation
Focus Group	F	63	5–6	28.9	Housewife
Focus Group	F	62	6	32.4	Housewife
Focus Group	F	58	6–10	28.9	Housewife
Focus Group	F	60	7–10	27.7	Primary healthcare centre administrative assistant
Focus Group	F	45	8–10	29.7	Head of services in the Town Hall
Focus Group	F	48	6–14	29.2	Unemployed administrator
Group 1	F	58	6–7	27.8	Translator
Group 1	F	56	6–10	28.2	Caregiver in a residence for the elderly
Group 1	M	57	9	27.8	Computer programmer
Group 2	F	58	5–6	30	Housewife
Group 2	M	59	11	28.8	Pharmacy laboratory technician
Group 3	F	58	6–10	28.9	Primary healthcare centre administrator
Group 3	M	45	6–10	27.3	Administrator
Group 3	M	47	6–10	31.4	Civil servant
Group 3	M	56	6–10	33.3	Treasury official
Group 4	F	54	6–10	34.8	Unemployed
Group 4	F	62	6–10	29.0	Retired
Group 4	M	48	6–10	30.8	Primary healthcare administrative assistant
Individual 1	F	41	6–10	26.0	Primary healthcare centre administrator
Individual 2	F	54	8	25.2	Director and teacher at a primary school
Individual 3	M	52	6–10	29.4	Administrative department head
Individual 4	M	34	6–10	30.0	Resident in family/community medicine
Individual 5	F	25	6–7	31.2	Student

### Data collection methods

Conversational methods were used to collect data in five group sessions and five semi-structured, in-depth, individual interviews. Initially, focus groups were planned, but the limited number of participants (from 2 to 4) in four of the groups led to a decision to form four triangle groups and one focus group (Table 2).

**Table 2:** Interview schedule.

**1. Activities carried out while sitting**
**a.** Opinion and beliefs about being seated
**b.** Main activities (work, free time/weekend)
**2. Willingness to reduce sitting time**
**a.** Importance, perception of the need, benefits and inconveniences
**b.** Motivation and confidence
**3. Determining factors and suggestions for change**
**a.** Difficulties and barriers
**b.** Aids and motivators
**c.** Suggestions
**d.** How to make a change
**4. Views on help from the primary health care centre**
**a.** How participants thought primary health care could help
**b.** Follow-up that participants would want from the primary healthcare centre

In addition, individual interviews with five patients who met the study criteria were carried out as a pilot study in order to identify topics to be explored and design the interview script for the present study. These topics included opinion and beliefs about the time spent sitting and activities carried out while sitting; willingness to reduce the time spent sitting (importance, motivation, confidence); barriers, facilitators and suggestions; and views on support from primary healthcare professionals (Table 2).

### Fieldwork

Recruitment of participants and group and individual interviews took place between January and July of 2012. All interviews were carried out at the participant’s assigned primary healthcare centre, but away from the usual office visit environment. Group interviews were moderated by an expert in qualitative investigation and included an observer; both of them were unfamiliar to the participants. Interviews lasted between 60 and 90 minutes.

### Analyses

All interviews were taped and transcribed systematically, literally, and anonymously. An analysis was made of thematic content, coding the data and grouping them into predefined categories based on the interview topics. The analysis was done with the support of Atlas.Ti and by triangulation of analyst.

## Results

The study included 23 participants (15 women and 8 men), mean age 52 years (range, 25–64), with a body mass index (BMI) of 29.4± 4.8 kg/m^2^, and who spent 6 to 14 hours a day sitting. Detailed characteristics are shown in Table 1.

Three occupational profiles were developed, according to the following sedentary behaviours:

**Sedentary workers (administrative offices and/or public information):** Individuals spend most of the day sitting because of the needs of the job, with tasks that depend mostly on computer use.

**Housewives and retired people:** Individuals who usually do household tasks and errands in the morning but generally spend many hours in the afternoon and evening doing sedentary activities such as reading or sewing.

**Continuing education participants:** Individuals who spend many hours sitting in classes, where normally they do not move around, and are also sedentary at home, studying and working on a computer.

Results for the list of interview topics (Table 2) are presented below, and associated with the relevant occupational categories.

### 1. Sedentary behaviour: opinion and main activities

Participants demonstrated difficulty in talking about the time that they spent each day sitting, focusing more on explaining whether they did or did not do enough physical exercise. Sedentary behaviour was understood as normal and was seen as good because they liked it and it gave them comfort, especially when they were tired, whether physically or mentally.

The main activities carried out while sitting were:

-Using the computer (checking emails, looking for information on the Internet) at work, for study, and at home

*I am one of those people that have to spend many hours sitting because of the nature of my work; I am a computer programmer*. (Man, 57 years old, computer programmer; Triangle group 1)

-Sedentary work and continuing education, where the individual must remain seated in class and also while studying at home

*Lately*, *because I don’t have work*, *I have signed up for classes*, *and I am doing a Masters degree at the University*, *spending consecutive 6 hours sitting*… *Then*, *I am at home in the mornings*, *sitting at the computer looking for work*. (Woman, 54 years old, unemployed; Triangle group 4)

-Journeys, both for work and apart from work, in own vehicle or on public transport.

I always drive to work because I live far away. (Man, 57 years old, computer programmer; Triangle group 1)

-Other activities such as watching television, eating or sitting at the table, reading, sewing or crocheting.

The majority maintained these sedentary activities on workdays and also during free time at the weekend, although some mentioned that they made an effort to do some sort of physical activity during the weekend.

*Well*, *we try at the weekend*, *to get out and walk*, *or go cycling or go to the swimming pool or something*. *We make an effort*, *we are aware that during the week we cannot do anything*. (Woman, 54 years old, director and teacher at a primary school; Individual interview)

### 2. Willingness to make a change

#### 2a Importance (perception of need, benefits, and drawbacks)

-In general, participants were not conscious of passing much time sitting, because they did not give it much importance. The majority had not thought about reducing this behaviour.

*I thought that I didn’t spend much time sitting*.... *when really it adds up to a lot of hours… you realise that it is much more than you thought*. (Woman, 54 years old, director and teacher at a primary school; Individual interview)

-Participants did not know the negative health consequences (cardiovascular disease or mortality) of remaining seated for a long period, although they did relate prolonged sitting to short-term negative effects. They described physical effects such as poor circulation, with heaviness and pins and needles in the legs; aching back, muscles and joints; less flexibility and an increase in weight. On the emotional level, participants explained that it generated discomfort, bad temper and “mental tiredness”, but not physical tiredness, and made it more difficult to get a good night’s rest.

*I also sometimes have problems with bad circulation and I am aware of tired feet*. *And*, *when I am on holiday or when I have the opportunity to walk more*, *that doesn’t happen*. (Woman, 54 years old, director and teacher at a primary school; Individual interview)

-Participants thought that they should avoid sitting for many hours in succession to eliminate these effects. In addition, in their opinion, these sedentary behaviours generated other habits that are bad for health (posture, snacking between meals) and establish a vicious circle whereby the more they sit the less they feel like doing non-sedentary activities and the less agile they become.

*And being sedentary brings you to that*, *to have less and less interest in doing anything*, *and it is dramatic* (Man, 47 years old, civil servant; Triangle group 3).

#### 2b Motivation and confidence

During the interviews, after the comments on the negative consequences of prolonged sitting, participants showed an interest in reducing the time they spent sitting, but saw difficulty in exchanging sedentary routines, which are comfortable and involve little effort, for more active habits.

*Let’s see*, *I don’t feel bad sitting down*. *If I am involved in something*, *it’s better*, *but I understand that it isn’t good*, *that one should move more*. (Woman, 58 years old, housewife; Triangle group 2)

For change to occur, participants believed it necessary to have appealing alternatives that they would enjoy and that would motivate them. The majority had little confidence in achieving change, above all at work.

### 3. Determining factors and suggestions for change

#### 3a Difficulties and barriers to reducing sitting time

The lack of awareness of time spent sitting and of the negative consequences for health, along with the effortless nature of a comfortable habit, make it difficult to consider making changes:

..*to be sitting all day*, *in principle*, *isn’t a bother*, *it is a way of life*…, *nor is it that you live badly because you are seated all day*. (Man, 57 years old, computer programmer; Triangle group 1)

…*you think about it but in the end you don’t do it*. *I don’t know whether it is because we always have other goals*, *there are always other things to do so that in the end you put aside the walk; you start being comfortable again and soon you end up always sitting down* (Woman, 54 years old, unemployed; Triangle group 4)

The main difficulties that were identified or listed during the interviews are described below and summarized in Table 3:

**Table 3:** Main barriers to reducing sitting time.

Barriers to reducing sitting time
• Lack of awareness of sitting for much time and of its consequences
• Perception of well-being while sedentary, and acquired habits
• Work routines: sedentary work, dependence on technology, corporate culture andeducational culture
• Family routine and obligations (responsibilities)
• Lack of time: daily pace of life (hurry to get to places)
• Influence of sedentary friends and family
• Age: The older one gets, the more difficult it is to change any routine
• Lack of motivation, of willpower
• Physical tiredness and especially mental tiredness
• Lack of acceptance for activities subject to a timetable (they preferred activities done at will, for example, on holiday)
• Passive leisure activities: videogames, cinema, television

-Work routines, for those where the surroundings (type of desk, customer service at a counter, and/or dependence on a computer) require that the worker be seated and where this is the most comfortable way of working:

*At work these days everyone is sitting*, *almost all at a computer*. (Woman, 54 years old, unemployed; Triangle group 4)

On the other hand, the business culture also has well-established norms of conduct, according to which the employees should be sitting during their working hours.

*Something that you could do was some exercises*, *stretching*, *but because it isn’t normal if you do it everyone else looks at you as if you are eccentric*. (Woman, 48 years old, unemployed administrator; Focus group)

The same happens in the context of education, given that it is not seen as good if the students are standing up. From a very early age, we learn–and become accustomed–to spend many hours sitting.

*Since I was little*, *in school*, *you also sat down for many hours so you become used to living like that*. (Man, 57 years old, computer programmer; Triangle group 1)

-The daily routine and family obligations make it difficult to have time free for less sedentary activities.

*…the family also pushes you*. *Therefore*, *you spend all day at work sitting*, *you get home*, *…always sitting there*, *you can't move*…, *waiting to see if your child will or won’t arrive late*, (Man, 45 years old, administrator; Triangle group 3)

-The lack of time and the daily pace of life, hurrying to arrive places, together with the distances involved, favour travelling by public or private transport, in which one is also seated.

*The issue is always the same*, *it is lack of time*, *because you are in a hurry*, *and you are always in a hurry*, *you take the car*. *You could walk*, *but you want to do several things and you end up taking the car*. (Woman, 54 years old, unemployed; Triangle group 4)

-Sedentary friends and family have an influence. If the family and surrounding friends are mostly sedentary, it will be difficult to make changes in the way free time is spent.

*The fact is that on Saturdays I go on foot with my mother*, *but if I go with my father*, *we go by car because he doesn’t want to walk*. (Woman, 25 years old, student; Individual interview)

-Increasing age has repercussions at the physical level and makes it more difficult to have a life as active as when younger, and more difficult to change habits

*The older we become*, *the body becomes a little more sluggish*. *One becomes lazier and you realise that it takes more effort to change your habits and become more active*. (Man, 59 years old, pharmacy laboratory technician; Triangle group 2)

-Lack of motivation or willpower can be a factor. Housewives, in particular, commented that one fell into a daily routine or vicious circle that made them increasingly sluggish, which then required more effort to do any physical activity. Many didn’t like exercise and said that it made them tired. In the end they opted for the maximum comfort, doing more sedentary activities.

*I have no will power*, *I would have to be forced*, *ordered; if not*, *on my part*, *no*. (Woman, 63 years old, housewife; Focus group)

-The physical tiredness and above all mental fatigue experienced after a day’s work, even if the work is sedentary, make it difficult to do any physical activity and favour sedentary behaviour after arriving home.

*After work*, *you always finish mentally tired*. *You arrive home and you stretch out and have no desire to move; what you want most is to get comfortable*. *It is a mental tiredness because clearly you haven’t done anything physical during the day*. (Man, 57 years old, computer programmer; Triangle group 1)

-Leisure time activities that are passive like video games, cinema and television do not require any movement or physical effort.

*My co-worker is 60 years old*, *I asked him*, *“What have you been doing for 4 days stuck in the house*?*”*. *He said*, *"Nothing*, *playing on the PlayStation"*. *Can you believe that he spent 4 days at a stretch playing*, *a person who is 60 years old*? *No*? *Because we think it is a hobby for the young*. (Man, 45 years old, administrator; Triangle group 3)

#### 3b Aids and motivators for reducing sitting time

The facilitators that people mentioned during the interviews are detailed in Table 4, with the following examples:

**Table 4:** Main facilitators to reduce sitting time.

Aids for reducing the time spent sitting
• Active and helpful family environment
• Good climatic conditions (change easier in summer)
• Holidays and free time (availability of time)
• Need to move and the well-being that results from activity (feel better after doing non-sedentary activities).
• Social and work changes that allow a change in the pace of daily life

-An active and supportive family environment that made it easier for someone to think that they should spend less time sitting and consider doing more physical exercise.

*Every now and then my sister offers to go with me somewhere and so*, *that afternoon*, *I don’t even remember that there is a sofa and the afternoon passes*, *but afterwards*, *by my own effort*, *no*, *if I am not motivated*, *no*. (Woman, 58 years old, housewife; Triangle group 2)

-Good climatic conditions with more daylight hours and pleasant temperatures encourage people to go out and walk more and not spend so much time at home, sitting.

*You wait for it to be a little warmer to go out more*, *walk more*, *take the bicycle more*. (Woman, 58 years old, translator; Triangle group 1)

-Holidays are when there is more free time available to carry out non-sedentary activities that people enjoy and cannot do during the rest of the year.

*I enjoy walking; I even get up earlier when I am on holiday than when I am working*. *I do it with enthusiasm*. *Nobody makes you*, *you are doing it because you want to*, *you know that you have time and that you don’t have to follow the daily routine that you do all year*. (Man, 48-year-old administrator of a primary healthcare centre; Triangle group 3)

-Feeling a need to move and the wellbeing that results from activity is another motivator. Participants move more "because the body demands it" and for the well-being that results, rather than focussing on the potential health benefits.

*But I do this out of habit*, *not because I think it is better; it’s because the body asks for it*. *Instead of sitting all the time*, *I will go and walk for awhile*. (Man, 57 years old, computer programmer; Triangle group 1)

-Changes socially or at work can allow a change in the pace of daily life and the possibility to adapt to the needs of family and recreation, resulting in more free time.

*Maybe it depends on your profession*, *but I think that the workdays are too long*. *The ideal would be a shorter workday*, *with more free time for family recreation* (Woman, 58 years old, translator; Triangle group 1)

#### 3c Suggested changes for reducing sedentary time

Especially notable was the difficulty that participants had in thinking of and suggesting specific changes. In general, they thought it would be easier to reduce the amount of time spent sitting by doing other activities that involved movement, rather than to stand up to do activities normally done while sitting down (eat, drink coffee, watch TV, use the computer).

*I go out to see something or to buy something*, *yes*, *but to be standing at home and just be still*, *that would be very tiring*. *To eat or watch TV standing up*, *well no*. (Woman, 58 years old, housewife; Triangle group 2)

The main suggestions according to occupational profile, type of sedentary behaviour, and lifestyle were the following:

**People with sedentary work, administrative and/or dealing with the public:** The most feasible alternatives were to stand up more often (set some rules to force yourself to stand up every so often); to stand up during rest periods or wander around; and to alternate tasks that are done sitting with those that can be done standing up.

**Housewives and retired people:** The main problem was a lack of willpower. Participants commented that they ought to use their free time for less sedentary activities that they enjoyed, either alone or in a group.

**People in continuing education:** As alternatives, participants suggested standing up more often, at fixed intervals; making an effort to read or study while walking around; and using classroom breaks as a time to stand up

*Yes*, *between classes when we go out into the corridor*, *we always sit on the benches (laughs)*. *So*, *probably*, *we should be standing up*. (Woman, 25 years old, student; Individual interview)

During the interviews, the following suggestions were made (see Table 5):

**Table 5:** Main suggestions for reducing sitting time.

Suggestions for reducing sitting time
**1. At work**
**a.** Stand up every now and again: to drink water, smoke, speak on the phone, communicate with colleagues
**b.** Rest time: walk, go up and down stairs
**c.** Make journeys on foot
**2. At home**
**a.** Do the ironing standing up (while watching television)
**b.** Get up during the advertisements (do jobs)
**c.** Go out for a walk instead of spending more time at home
**d.** Do-it-yourself or jobs around the house
**e.** Put on music and dance
**3. Leisure time**
**a.** Take the dog out for a walk
**b.** Play or go to the park with the children
**c.** Go out to walk in the commercial centres and markets
**d.** Watch television or read on a static bicycle
**e.** Play with the WI (or similar)
**4. Journeys**
**a.** Go by foot or by public transport
**b.** Reduce the use of public transport to the essential; do the rest on foot
**c.** Public transport: get on further along the route or get off earlier
**d.** Use the stairs instead of the elevator

### At work

Although many participants had to be sitting down to carry out their habitual work, such as using the computer or dealing with the public, they suggested trying to stand up more often and to do more activities on foot or walking.

*…me too*, *within whatever has to be done sitting*, *I will make sure that I stand up when I can*. (Man, 45 years old, administrator; Triangle group 3)

- Stand up often to drink water.
- Intersperse tasks that are done on foot with those that have to be done sitting down.
- Move and communicate in person with work colleagues if possible, instead of using the phone or emailing.
- Have work meetings with other colleagues on foot or taking a stroll.
- Stand up when talking on the phone.

*Usually*, *I stand up when I am talking on the phone*. *I speak standing up straight*, *and also walking*… (Man, 52 years old, administrative department head; Individual interview)

- Set up work rooms such that it is possible to work for a little while standing up at the computer.

*The changes should be to set up rooms for working on foot and*, *depending on the task*, *instead of putting a table*, *put counters with computers so that*, *it would allow you to move*. (Man, 52 years old, head of department–administrative; Individual interview)

- In rest time, walk, go up and down stairs or remain standing; avoid having breakfast or eating at the work desk.
- Make the journey to work on foot.

*The first thing that you have to note down is to walk to work*, *don’t take the lift*, *go up the stairs; many small things that if you add them up I am sure would make a difference throughout the day*. (Man, 47 years old, civil servant; Triangle group 3)

### At home

- Do jobs around the house while standing: ironing, washing dishes, hanging out the washing, or do-it-yourself projects, for example.

*At home I don’t sit very much because it is easier to fold clothes or iron standing* up. (Woman, 54 years old, director and teacher at a primary school; Individual interview)

- Get up during the adverts or to change the channel on the TV.
- Put on music and dance.

### In leisure time

- Walk the dog. If you have a dog, it creates the obligation to take it out for walk every day

*…*.*but what saves me is that I have two dogs and I have to take them out for a walk half an hour every day of walks at dusk*. (Man, 57 years old, computer programmer; Triangle group 1)

- Play and go to the park more often with the children.
- Walking to the shopping centre and market is a way of spending hours walking, especially for those that enjoy looking at shops and have to make an effort to go out walking.

*I can spend 4 hours walking in the shopping centre*, *looking at this and that*. *The afternoon passes very quickly*. (Woman, 58 years old, housewife; Triangle group 2)

- Look for non-sedentary activities to fill free time.

### In journeys

- Make trips on foot and keep the use of motorized transport to a minimum. This was one of the suggestions that the participants thought most feasible, both for work and during leisure time. They suggested achieving this by taking public transport or your own vehicle only when necessary because of distance or lack of time.
- If using public transport, stay standing.

*On public transport*, *which I use every day*, *I try to stand and not sit down*. (Man, 52 years old, administrative department head; Individual interview)

- Travel less distance on public transport and do the rest on foot. If you use public transport, try to get off before your destination or to get on later.

*I also try*, *instead of taking the metro*, *which is very close*, *I walk to catch the tram*, *which means that I walk for longer*, *perhaps a quarter of an hour or 20 minutes walking*. (Man, 57 years old, computer programmer; Triangle group 1)

#### 3d How to make changes

- The majority of participants preferred to do non-sedentary leisure activities with a group, because they were more enjoyable and motivated them more.

*Well*, *for me*, *to do something with other people is always more pleasant some things are done better in company—sports*, *especially* (Man, 57 years old, computer programmer; Triangle group 1)

Although some preferred to do such activities alone:

*I love to do sports and don’t need anyone*. *Yes*, *I have the custom*, *both in sport and at work*, *of being alone; I don’t need anyone to encourage me*. (Man, 59 years old, pharmacy laboratory technician; Triangle group 2)

- Do physical activities in the open air. Many were bored in the gym.
- Use information and suggestions at the social and media level to change this behaviour, especially during childhood, by educating people to adopt less sedentary habits and to move about in the work environment.

*I think it’s a question of teaching and of habits*, *above all at an early age*, *when we learn everything*. (Man, 59 years old, pharmacy laboratory technician; Triangle group 2)

It would be advisable to make the public more aware, informing them about the ill effects of being seated for many hours. Publicity campaigns via various means of communication could be very useful, giving appropriate advice.

*A publicity campaign I believe would have a lot of influence because if it says “sitting for a long time can cause cardiovascular problems”*, *well*, *you have to spend less time sitting*… *I think that campaigns are the things that do the most to affect the way in which we live*. (Woman, 60-year-old administrator of a primary healthcare centre; Focus group)

At the work level, in order to implement some of these suggestions, companies should first become aware of the need for employees to sit less and of the resulting benefits. Regulations are needed, proposing work guidelines that make it easier for employees to stand up, at least sometimes, and to carry out some tasks on foot.

*if not by making rules*, *through advice*. *they could incorporate this theme to say*, *“in addition to being seated well*, *every so often you should stand up etc*.*” It would be normal and people wouldn’t see it as bad that they had to get up to get a glass of water or walk to the corner and back and no one would say “oh that person is skiving off”*. *It isn’t only that you should be aware but also that*, *bit by bit*, *the environment should help a little*. *The company should also be aware*, *especially the big companies*. (Man, 52 years old, administrative department head; Individual interview)

### 4. Views on help from primary healthcare professionals

#### 4a How do participants think that primary healthcare efforts could help them to spend less time sitting?

-Advice and suggestions from the professionals working in primary healthcare would be helpful. Participants believe that the doctors and nurses inspire confidence and could raise awareness and help people that spend a lot of time sitting to adopt more healthy habits. They could offer guidelines on doing exercise and stretching when many work hours must be spent sitting.

*Well*, *if the doctor tells you something*, *you usually take notice*. (Woman, 54 years old, unemployed; Triangle group 4)

-Participants opted for group interventions that include practical help (not only theory, and in addition to advice during office visits). They believe that a single visit to a primary healthcare professional in which some instructions to reduce sedentary behaviour are given is not sufficient to raise awareness and to break these habits. On the other hand, group activities allow participants to share experiences with others.

-Participants proposed interventions in groups that were homogeneous with respect to age or type of work, so that it would be possible to share similar experiences and learn new strategies to apply at work. In addition, group interventions create a group commitment that requires attendance.

*If a group is homogeneous at the level of age or work*, *and with a series of ideas to share*, *for example*, *she and I work similarly*, *then you can share*. (Woman, 45 years old, Town hall head of service; Focus group)

#### 4b What follow-up would participants want from primary healthcare professionals?

-Participants think that patient follow-up by email, or personal follow-up in office visits for those that don’t have email access, is important. A nurse could do this, in the same way that they monitor other activities such as diet and weight control.

*Yes*, *follow-up by email is the easiest; most people have it and use it during the day*. *whether people would look at it*, *that is another question*. (Man, 57 years old, computer programmer; Triangle group 1)

*At the level of the nurse*, *the same way that they carry out controls such as weight and blood pressure monitoring; well*, *the control of this*, *how it’s going*, *how much you are walking*, *that you are not spending too much time sitting … it would be taken into account*. *If every 3 months you visit the nurse*, *like for other controls*, *well*, *it could be a little push*. (Woman, 60-year-old administrator of a primary healthcare centre; Focus group)

-Participants suggested a follow-up after one to three months and some type of evaluation; for example, a questionnaire that evaluated the time spent each day sitting and monitored the progressive reduction.

*Well*, *by email would be good*. *Equally*, *it could be good to give out a type of questionnaire*, *this time more detailed*. *“Count for one day how many hours you are sitting while on the telephone*, *the computer or whatever” and after 2*, *4*, *6 months or whatever time*, *give out the same test and look to see whether there is any significant difference*. (Woman, 54 years old, director and teacher at a primary school; Individual interview)

Other relevant full-length quotes can be seen in S1 File.

## Discussion

### Summary of the main findings of the study

Our primary observation was that reducing the number of hours that overweight or obese people spend sitting requires interventions that are feasible, practical, acceptable and effective. Therefore, it is necessary to understand sedentary behaviour and include the opinions and suggestions for improvement given by the target population.

The major findings of this study are:

1. Participants expressed a lack of awareness about the time spent sitting and did not know about the negative health consequences, especially over the long term. Sedentary conduct was understood as normal and, although there was interest in reducing it, they envisioned difficulty in changing their habits. For change to happen, suggestions are needed for attractive alternatives that they would enjoy and that would motivate them. In addition, the majority had little confidence in being able to achieve change, above all in the work environment.
2. The most usual activities that are carried out while seated are work and study in front of a computer. At home and during free time, the computer and watching television were mentioned most of all. Also, most journeys were made sitting in a private vehicle or in public transport.
3. Highlighted difficulties in changing this behaviour were family and work routines, lack of time, and the distance travelled. Sociocultural barriers were also described, along with a lack of willpower, tiredness after a working day, and the difficulties that accompany increasing age. In contrast, factors that help reduce sedentary behaviour include feeling emotionally and physically better after being more active; a close environment of family and friends who are active and helpful; free time; and a good climate.
4. Changes are needed at the social level, using publicity campaigns in corporate culture and in the sphere of education, with regulations and guidelines that encourage and allow a reduction in the time spent sitting.
5. Participants did not consider standing while carrying out activities that they normally did while sitting, but rather suggested a need to move more.
6. Professionals at the primary health care centre should inform, raise awareness, and help patients to adopt habits that reduce sitting time. Participants preferred group interventions with practical support and groups that are homogeneous in age and type of work. It is important that some follow-up be provided, either in person or by email. Monthly follow-up was recommended, during which some sort of evaluation is made, such as a questionnaire that assessed the time spent sitting and any reductions achieved.

### Comparison of the study with others in the literature

The physical, social and economic changes produced in our society have reduced our physical activity and increased the time we spend sitting. Multiple elements have an influence on these behaviours, including individual factors such as beliefs, preferences, and motivations and familial, sociocultural and other factors in our home, work, and leisure environments.

We live in an environment that requires us to be seated for prolonged times [2]. According to our study, most of the hours spent sitting take place at work and while studying, above all in front of the computer. This finding coincides with that of Owen [3], who noted that the main reason adults maintain a seated position for long periods of time was employment in activities that involve sitting. Gilsen et al. [21] analysed the views of employees on the health risks of the time spent sitting in the workplace and suggested intervention strategies to interrupt or reduce this sedentary time.

Other environments in which people spend many hours sitting include their leisure time at home and their movements in private vehicles or public transport. Again, our study coincides with those of Owen [3] and Dunstan [2] in that the main activities reported in the domestic environment are watching television, using a computer, and other recreational screen time. A large proportion of the data on sedentary time has been obtained from studies of time spent watching television. An American study [31] associated watching 4 or more hours of television a day with a lower level of education and with obesity.

With respect to journeys in private vehicles, which provide no alternative to remaining seated, various factors could have an influence, such as place of residence, living in areas that are remote or with little infrastructure, the distance and accessibility of the workplace, and the existing network of public transport [32].

Other studies have analysed barriers and facilitators but focused more on physical activity. Suggs [33] looked at a younger group of sedentary people (25–35 years old) who were overweight or obese. A study by Niñerola [34] analysed the barriers perceived by university students and users of sports clubs, mentioning laziness, lack of willpower, and tiredness during exercise. Both of these studies noted work obligations, family obligations, and lack of time as the main barriers for most participants.

In the study by Matthews [5], increased age was also highlighted as an important difficulty. Young adults aged between 20 and 29 years were the most active, whereas sedentary activities increased for both sexes in the group aged 30 to 39 years. The age group ranging from 70 to 85 years was the most sedentary, with women and men sitting for 9.1 and 9.5 hours a day, respectively.

Regarding facilitators, our results coincide with the study by Suggs [33] in identifying exercise with other people and good weather as helpful elements. Participants believed social norms that encourage physical activity are necessary, with a greater political involvement by the government. Some television programs have been able to motivate these participants to do more exercise.

Among the suggestions of ways to reduce the time spent sitting at home, our study participants mentioned getting up more often, for example during television adverts or to change the channel manually instead of using a remote control. A clinical trial in the U.S. carried out a three-week intervention to reduce TV viewing in an overweight/obese population aged 22 to 61 years, similar to our study participants. The intervention group achieved a reduction of 61% (3.8% of all sedentary time) [34].

In the workplace, our study participants suggested moving around more frequently and doing more tasks while standing or walking; they also suggested equipping workspaces with standing computer desks. The literature contains very few randomized, controlled clinical trials that evaluated the impact of interventions designed to reduce the number of daily sedentary hours in the work setting [21–23]; one of these trials [23], involving 12 patients diagnosed with overweight and obesity, showed that the use of treadmill workstations at the workplace increased minutes of walking time per day and number of steps taken per day, and decreased sitting/lying time.

Assessment and follow-up by primary healthcare professionals, whose advice and suggestions could help reduce sitting time, has also been highlighted as necessary. In our study and that by Suggs [33], patients considered the work of these health professionals to be important in promoting healthy habits such as physical exercise or, in the case of our study, reducing sedentary behaviour.

In contrast, primary healthcare professionals viewed such promotional activities as having little effect and saw a lack of integration with their work routine [35]. Suggs [33] found that 50% of doctors did not encourage overweight and obese patients to increase their physical activity. In Catalonia, 88% of health care professionals reported an irregular promotion of physical activity for their patients, using generalized messages and only for certain patients [36].

### Limits of the study

Certain limitations are inherent in the study design, given the subjectivity of the research team at all phases of the study (literature search, design of the interview scripts, and analysis of the results). Nonetheless, the availability and flexibility of the research team and of an expert in qualitative methods, the pilot study conducted to develop the interview script, the literal and systematic transcription of the interviews, and the triangulation of analysts all contributed to control this effect.

Participant recruitment was difficult, let alone the selection of a diverse sample. We invited 30 patients to participate, of whom 23 finally attended the interviews. Although we had planned to have five focus groups, in the end we made four triangle groups and one focus group. Possible explanations for the difficulties in recruitment were the lack of time and that the interviews were conducted in the health centre at fixed times, which made participation difficult if something unexpected happened. The study by Suggs [33], in a similar population, also had few participants.

One of the limitations of the study, as well as in other qualitative studies that use convenience sampling, is that participants that accept to participate could be those who are more aware of the risks of prolonged sitting time or those who have a stronger feeling regarding changing sitting habits. Although the sampling was based on pragmatic criteria, of feasibility and of accessibility, to ensure the widest variety of discourses on sedentary behavior, selected participants were of both sexes, of different ages and occupations, and had different levels of overweight and control their obesity. These are the key characteristics of the population included in the SEDESACTIV clinical trial and the speeches are representative and potentially transferable to populations of similar characteristics. In addition our study was saturated with information from group and individual interviews (triangulation of methods), so that the information obtained fulfils the dual criteria of convenience and sufficiency.

The sample selected (aged between 25 and 65 years, overweight or moderately obese, and receiving primary health care) does not allow us to transfer our results to other populations. The sample predominantly included females (65%) and older individuals (only 4 participants were 45 or younger), and it is unknown whether the views expressed in this study are subject to any bias as a result of these characteristics. Consequently, is probable that the results are less applicable to this specific age group. Further studies should analyze in depth the opinions and experiences of this specific group. However, in Spain, those individuals who frequently attend primary care centers tend to be the older (and who suffer from illnesses treated in primary care). Nonetheless, the sample proved to be very useful for the design of interventions aimed at reducing the amount of sitting time for this profile of primary healthcare patients.

### Strong points and relevance for daily practice

Our study is relevant from the point of view of daily practice, because it explores the opinions of overweight or moderately obese people about a behaviour that is becoming increasingly more common, is detrimental, and has not been well studied. The contributions of the participants were of great use in designing the SEDESTACTIV interventions such that they would be more feasible, practical, and effective for this population. The opinions of those participating in group interventions during the SEDESTACTIV study could stimulate another qualitative study to analyse their contribution to what is known about barriers, facilitators and motivation to change sedentary behaviours.

This study could also be useful for the design and evaluation of future interventions to reduce sedentary behaviour in other profiles (other ages or those of normal weight).

## Conclusions

Considering the data contributed by the study, we consider it necessary to raise the awareness of primary healthcare patients concerning the importance and possible health benefits of reducing the amount of sitting time. Similarly, it is necessary to assess the main difficulties and barriers to changing this behaviour and for the primary healthcare centre to provide alternatives and appealing suggestions, both individualized and in group settings. These interventions should be guided and monitored over time by primary healthcare professionals. It is also necessary to make changes in social and work settings that can favour a reduction in sitting time. The qualitative data obtained by the present study was used to design the randomized controlled trial (RCT) using an education-based intervention to reduce sitting time in the SEDESTACTIV project which aims to assess people’s understanding health risks derived from excessive sitting time and to test the effectiveness of an education-based intervention to reduce sitting time. The intervention includes information on the importance of reducing sitting time and its health benefits. It also offers alternatives to prolonged sitting time in the personal, working and travelling environment proposed by the participants in the present qualitative study.

## Supporting Information

S1 File This is the S1 File. Other barriers and facilitators to reduce sitting time.
(DOC)

## References

[1] Network SBR (2012) Letter to the editor: standardized use of the terms “sedentary” and “sedentary behaviours”. Appl Physiol Nutr Metab 37:540–542. 10.1139/h2012-024 22540258

[2] Dunstan DW, Howard B, Healy GN, Owen N (2012) Too much sitting–a health hazard. Diabetes Res Clin Pract 97: 368–376. 10.1016/j.diabres.2012.05.020 22682948

[3] Owen N, Bauman A, Brown WJ (2009) Too much sitting: a novel and important predictor of chronic disease risk? Br J Sports Med 43:81–83. 10.1136/bjsm.2008.055269 19050003

[4] Healy GN, Wijndaele K, Dunstan DW, Shaw JE, Salmon J, Zimmet PZ, et al (2008) Objectively measured sedentary time, physical activity, and metabolic risk: the Australian Diabetes, Obesity and Lifestyle Study (AusDiab). Diabetes Care 31:369–71. 18000181 10.2337/dc07-1795

[5] Matthews CE, Chen KY, Freedson PS, Buchowski MS, Beech BM, Pate RR, et al (2008) Amount of time spent in sedentary behaviors in the United States, 2003–2004. Am J Epidemiol 167(7): 875–881. 10.1093/aje/kwm390 18303006 PMC3527832

[6] Farinola M (2011) Conducta sedentaria y salud: Antecedentes y estado actual de la cuestión. Red Nacional de actividad física y desarrollo humano. Año I No 95. 20 de diciembre de 2011.

[7] Van Uffelen JG, Wong J, Chau JY, van der Ploeg HP, Riphagen I, Gilson ND, et al (2010) Occupational sitting and health risks: a systematic review. Am J Prev. Med 39: 379–388. 10.1016/j.amepre.2010.05.024 20837291

[8] Wilmot EG, Edwardson CL, Achana FA, et al (2012) Sedentary time in adults and the association with diabetes, cardiovascular disease and health: systematic review and meta-analysis. Diabetologia 55(11):2895–905. 10.1007/s00125-012-2677-z 22890825

[9] Thorp AA, Healy GN, Owen N, et al (2010) Deleterious associations of sitting time and television viewing time with cardiometabolic risk biomarkers: Australian Diabetes, Obesity and Lifestyle (AusDiab) Study 2004–2005. Diabetes Care 33(2):327–334. 10.2337/dc09-0493 19918003 PMC2809275

[10] World Cancer Research Fund/American Institute for Cancer Research (2007). Policy and action for cancer prevention Food, nutrition, physical activity, and the prevention of cancer: a global perspective. Washington DC: AICR.

[11] Patel A, Bernstein L, Deka A, Spencer H, Campbell P, Gapstur S, et al (2010) Leisure time spent sitting in relation to total mortality in a prospective cohort of US adults. Epidemiology Research Program, American Cancer Society. Am J Epidemiol 172(4):419–429. 10.1093/aje/kwq155 20650954 PMC3590043

[12] Ploeg HP, Chey T, Korda RJ, Banks E, Bauman A (2012) Sitting time and all-cause mortality risk in 222 497 Australian adults. Arch Intern Med 172(6):494–500. 10.1001/archinternmed.2011.2174 22450936

[13] Katzmarzyk P, Church T, Craig C, et al (2009) Sitting time and mortality from all causes, cardiovascular disease, and cancer. Med Sci Sports Exerc 41, 998–1005. 10.1249/MSS.0b013e3181930355 19346988

[14] Pate R, O’Neill J, Lobelo F (2008) The evolving definition of “sedentary”. Exerc Sport Sci Rev 36:173–8. 10.1097/JES.0b013e3181877d1a 18815485

[15] Teychenne M, Ball K, Salmon J (2012) Promoting physical activity and reducing sedentary behavior in disadvantaged neighborhoods: a qualitative study of what women want. PLoS ONE 7(11): e49583 10.1371/journal.pone.0049583 23166718 PMC3498192

[16] De Heer H, Wilkinson AV, Strong LL, Bondy ML, Koehly LM (2012) Sitting time and health outcomes among Mexican origin adults: obesity as a mediator. BMC Public Health 12:896 http://www.biomedcentral.com/1471-2458/12/896 10.1186/1471-2458-12-896 23092387 PMC3527190

[17] Thorp AA, McNaughton SA, Owen N, Dunstan DW (2013) Independent and joint associations of TV viewing time and snack food consumption with the metabolic syndrome and its components; a cross-sectional study in Australian adults. IntJ Behav Nutr Phys Act, 10(1):96 http://www.ijbnpa.org/content/10/1/96 10.1186/1479-5868-10-1 23927043 PMC3751141

[18] Tudor-Locke C, Brashear MM, Johnson WD, Katzmarzyk PT (2010) Accelerometer profile of physical activity in normal weight, overweight and obese US men and women. IntJ Behav Nutr Phys Act 7: 60. 20682057 10.1186/1479-5868-7-60 PMC2924256

[19] Scottish Intercollegiate Guidelines Network (2010) Management of obesity. anational clinical guideline Edinburgh: SIGN Available: http://www.sign.ac.uk/pdf/sign115.pdf. Accessed 17.01.2011.

[20] Stevens J, Truesdale KP, McClain JE, Cai J (2006) The definition of weight maintenance. Int J Obes 30: 391–99. 16302013 10.1038/sj.ijo.0803175

[21] Gilson ND, Puig-Ribera A, McKenna J, Brown WJ, Burton NW, Cooke C (2009) Do walking strategies to increase physical activity reduce sitting in workplaces: a randomized controlled trial. IntJ Behav Nutr Phys Act 6: 43–50. 19619295 10.1186/1479-5868-6-43 PMC2717045

[22] De Greef KP, Deforche BI, Ruige JB, Bouckaert JJ, Tudor-Locke CE, Kaufman JM, et al (2011) The effects of a pedometer-based behavioural modification program with phone support on physical activity and sedentary behaviour in type 2 diabetes patients. Patient Edu. Counseling 84(2):275–279. 10.1016/j.pec.2010.07.010 20732776

[23] John D, Thompson DL, Raynor H, Bielak KM, Bassett DRJ (2010) Effects of treadmill workstations as a worksite physical activity intervention in overweight and obese office workers. Med Sci Sports Exerc 42 (5): 38. 10.1123/jpah.8.8.1034 22039122

[24] Otten JJ, Jones KE, Littenberg B, Harvey-Berino J (2009) Effects of television viewing reduction on energy intake and expenditure in overweight and obese adults: a randomized controlled trial. Arch Intern Med 169(22): 2109–2115. 10.1001/archinternmed.2009.430 20008695

[25] Gardiner PA, Eakin EG, Healy GN, Owen N (2011) Feasibility of reducing older adults’ sedentary time. Am J Prev Med 41(2): 174–7. 10.1016/j.amepre.2011.03.020 21767725

[26] Aadahl M, Linneberg A, Møller TC, Rosenørn S, Dunstan DW, Witte DR, et al. (2014) Motivational Counseling to Reduce Sitting Time: A Community-Based Randomized Controlled Trial in Adults. Am J Prev Med. 10.1016/j.amepre.2014.06.020 25113139

[27] Martín-Borràs C, Giné-Garriga M, Martínez E, Martín-Cantera C, Puigdoménech E, Solà M, Castillo E, et al (2014) Effectiveness of a primary care-based intervention to reduce sitting time in overweight and obese patients (SEDESTACTIV): a randomized controlled trial; rationale and study design. BMC Public Health 14:228 10.1186/1471-2458-14-228 24597534 PMC3973868

[28] Tong A, Sainsbury P, Craig J (2007) Consolidated criteria for reporting qualitative research (COREQ): a 32-item checklist for interviews and focus groups. Int J Quality Health Care 19(6): 349–357 17872937 10.1093/intqhc/mzm042

[29] Word Medical Association (2013). WMA Declaration of Helsinki–Ethical principles for medical research involving human subjects. Available: http://www.wma.net/en/30publications/10policies/b3/index.html 10.1001/jama.2013.281053 24141714

[30] Marshall A, Miller Y, Burton N, Brown W (2010) Measuring total and domain-specific sitting: a study of reliability and validity. Med Sci Sports Exerc 42(6): 1094–1102. 10.1249/MSS.0b013e3181c5ec18 19997030

[31] King AC, Goldberg JH, Salmon J, et al (2010) Identifying subgroups of U.S. adults at risk for prolonged television viewing to inform program development. Am J Prev Med, 38(1): 17–26. 10.1016/j.amepre.2009.08.032 20117553

[32] Owen N, Sugiyama T, Eakin EE, Gardiner PA, Tremblay MS, Sallis JF (2011) Adults’ sedentary behavior determinants and interventions. Am J Prev Med 41(2):189–196. 10.1016/j.amepre.2011.05.013 21767727

[33] Suggs S, McIntyre C, Cowdery J (2010) Overweight and obese sedentary adults physical activity beliefs and preferences. Am J Health Stud 25:69–77.

[34] Niñerola J, Capdevila L, Pinatel M (2006) Barreras percibidas y actividad física: el autoinforme de barreras para la práctica de ejercicio físico. Revista de Psicología del Deporte 15(1): 53–69

[35] Puig Ribera A, McKenna J, Riddoch C (2006) Physical activity promotion in general practices of Barcelona: a case study. Health Educ Res 21: 538–48. 16702195 10.1093/her/cyl008

[36] Puig Ribera A, McKenna J, Riddoch C (2005) Attitudes and practices of physicians and nurses regarding physical activity promotion in the Catalan primary health-care system. Eur J Public Health 15: 569–75. 16051654 10.1093/eurpub/cki045

