# Supplementary material for: Prolonged Sitting Time: Barriers, Facilitators and Views on Change among Primary Healthcare Patients Who Are Overweight or Moderately Obese
Source: PLoS One. 2015 Jun 9;10(6):e0125739. doi: 10.1371/journal.pone.0125739 (PMC4461272; doi:10.1371/journal.pone.0125739)
Supplement: S1 File — Other barriers and facilitators to reduce sitting time. (DOC) [file pone.0125739.s001.doc]

Supplementary file. Other barriers and facilitators to reduce sitting time

| **1b.3** | ... I’m always short of time. Just take the car, is what the others usually say. (Man, 45 years old, administrator; TG 3) |
| --- | --- |
| **1b.4** | *... and for all the activities, watching TV, the computer... usually all those are done sitting.* (Man, 57 years old, computer programmer; TG 1) |
| **2a.2** | *I get pins and needles in my legs, because it catches some nerves and I can’t endure it. When I am quiet watching television, then at the last minute, I realise the time that I have been wasting.* (Woman, 63 years old, housewife; FG) |
| **2a.3** | *And then, when you’ve been sitting more, it is also takes more effort to be more dynamic, more active*... (Man, 57 years old, computer programmer; TG 1) |
| **2b** | *In the end the sofa pulls me more than the gym, because it is also purely a question of laziness or of what, for me, is not appealing.* (Man, 56 years old, treasury official; TG 3)  *Yes, I would be interested, as long it didn’t affect me at my place of work* (Woman, 41-year-old administrator of a PHC; II) |
| **3a.1** | *It’s not that it doesn’t occur to me, it’s that there is no solution; you are in front of the public and have to deal with them while sitting at a computer.* (Woman, 60-year-old administrator, PHC; FG)  *I can’t change anything. I am in front of the computer, I have meetings (...) at work it would be difficult.* (Woman, 45 years old, Town hall head of services; FG)  *The work activity conditions you to be seated. I worked as a telephone operator, sitting for 6 hours at a stretch, always super-controlled [by supervisors]*. (Woman, 54 years old, unemployed; TG 4) |
| **3b.4** | *When I go to work by bicycle, I feel good, my legs don’t ache or anything.* (Woman, 58-year-old administrator of a PHC; TG 3) |
| **At work** | *But I don’t know, use the computer more standing up, more raised and do things standing* (Woman, 54 years old, unemployed; TG 4)  *... and to go downstairs for 5 minutes to have a cigarette... Now that I haven’t smoked for many years, well, I walk around for 5 minutes.* (Man, 52 years old, administrative department head; II) |
| **3d** | *Well, it should be something that is seen as normal for everyone and that it is normal at work to stand up and start to move, but it is a question of customs.* (Man, 57 years old, computer programmer; TG 1)  *What has to happen is a change in habits and in culture, so you would need to make a publicity campaign. But where it says what sitting causes, I haven’t seen that publicized and the first time that I’ve heard it is here.* (Woman, 45 years old, Town hall head of services; FG)  *The same as being told that you should go in at 7 in the morning, you could be told that every half hour you have to stand up for 5 minutes.* (Woman, 63 years old, housewife; FG) |
| **4a.1** | *(The doctors) always have knowledge and can guide you to things, and they know about other people in the same situation and can do this work of passing it on.* (Woman, 54 years old, director and teacher at a primary school; II) |
| **4a.2** | *By mail? The truth is that I don’t read much. But if there are talks, perhaps more people would be able to compare opinions and that would be good.* (Woman, 41-year-old administrator of a PHC; II)  *Groups always go well because you get new ideas and such.* (Woman, 25 years old, student; II) |
| **4b.1** | *Yes, follow-up by email is the easiest; most people have it and use it during the day.* (Man, 57 years old, computer programmer; TG 1) |
| **4b.2** | *Yes, I think the same, it could be good to make this type of evaluation, after a certain time, to see what behaviour you have adopted, how it has changed, whether you think that you have really changed the number of hours that you spend sitting*. (Woman, 54 years old, director and teacher at a primary school; II) |

TG: Triangle Group; FG: Focus Group; II: individual interview; PHC: primary healthcare centre
